# Temporal analysis of non‐communicable diseases and NCD‐HIV/AIDS comorbidity in Malawi: A 4‐year retrospective study 2020–2022

**DOI:** 10.1111/tmi.14134

**Published:** 2025-06-03

**Authors:** Ansley Kasambara, Mphatso S. Kamndaya, Salule J. Masangwi, Atupele Mulaga

**Affiliations:** ^1^ Department of Mathematical Sciences, School of Science and Technology Malawi University of Business and Applied Sciences Blantyre Malawi; ^2^ Centre for Water, Sanitation, Health and Appropriate Technology Development (WASHTED) Malawi University of Business and Applied Sciences Blantyre Malawi

**Keywords:** comorbidity, HIV/AIDS, machine learning, Malawi, NCD‐HIV/AIDS, non‐communicable diseases, XGBoost regression

## Abstract

**Objective:**

Non‐communicable diseases are on the increase in the Sub‐Saharan region, which is also the epicentre of HIV/AIDS. There is limited published data on trends and forecasts of the comorbidity of non‐communicable diseases and HIV/AIDS in low‐ and middle‐income countries. Hence, the aim of the study was to examine current trends and forecasts for non‐communicable diseases and non‐communicable disease‐HIV/AIDS comorbidity.

**Methods:**

Data on 30,686 patients from 2019 to 2022 were extracted from the non‐communicable diseases Mastercards, from 70 health facilities in Malawi, using a form designed and implemented in KoboToolBox. All cases were aggregated to form weekly counts of non‐communicable diseases and non‐communicable disease‐HIV/AIDS comorbidity and visualised using time series plots. Then the data was subset by the prominent non‐communicable disease, which was hypertension and hypertension‐HIV/AIDS comorbidity weekly counts. Extreme Gradient Boosting (XGBoost), a machine learning model, was used for model fitting, generating predictions, and forecasting.

**Results:**

The forecasts showed that the counts of cases per week will range from 137 to 185 cases. Then specifically, hypertension and hypertension‐HIV/AIDS comorbidity case counts averaged approximately within the range of 111–124 new cases per week from 2023 to 2030. The hypertension‐HIV/AIDS comorbidity had forecasts ranging from 2 to 9 new cases per week. Although the number of new case counts per week had a consistent progression, the number of cases was on an increasing trend over time. Therefore, whether cases in general, hypertension and hypertension‐HIV/AIDS comorbidity cases, or hypertension‐HIV/AIDS comorbidity cases, the counts per week imply cumulatively higher case counts.

**Conclusions:**

Despite the consistency in the projection to 2030, there is a need to consider the upward trend of these cases and implement intervention measures such as sensitisation to control the number of cases. Otherwise, Malawi may not be able to achieve SDG3 targets by 2030.

## INTRODUCTION

Trends in non‐communicable diseases (NCDs) are on the increase worldwide due to the surge in risk factors through urbanisation, industrialisation and globalisation [1, 2, 3, 4, 5]. Urbanisation, globalisation, and industrialisation have led to lifestyle changes; sedentary life and changes in diet which are risk factors of NCDs [3, 4, 6, 7, 8]. HIV/AIDS, on the other hand, has seen a decreasing trend in new infections globally [9, 10, 11]. Statistics show that there have been strides in the reduction of new infections with a few exceptional countries in Asia and Africa from which the trend is still increasing [10, 11, 12]. Due to the growing economic activities in some locations, these lead to high promiscuity propelling new HIV infections [4, 13, 14].

Low‐ and Middle‐Income Countries (LMIC), which include Sub‐Saharan Africa (SSA), have had an increase in the number of NCD cases [2, 7, 15, 16, 17, 18]. As a growing economy in SSA, Malawi has also experienced a rise in NCD cases [19, 20, 21]. Despite the trend in the reduction of new infections, the majority (65%) of HIV/AIDS cases are in SSA [10, 20, 22]. Malawi has observed a reduction in the number of new HIV cases by 75% since 2010, with an estimated 980,000 people living with HIV as of 2023 and a prevalence rate of 6.7% [23, 24]. Although these numbers highlight significant strides in the fight against HIV, risk factors are still prevalent in Malawi and SSA [25]. Therefore, HIV/AIDS is still an epidemic that needs commitment in implementing interventions to control and stop new infections [11, 12].

Location, and age of an individual are important characteristics in NCD and HIV/AIDS discussion. NCDs are prominent in specific locations. Likewise, HIV/AIDS infections tend to have high new infections in similar locations [26]. Cities and districts that possess a high and/or growing economy characteristic increase the risk of an individual having a NCD, HIV/AIDS, or NCD – HIV/AIDS comorbidity [27, 28]. NCDs were initially common among older individuals aged above 50 years old [19, 29]. This has somehow changed in recent years with young adults aged between 18 and 34 years old being afflicted with NCDs [30, 31]. HIV/AIDS is not age discriminant and therefore an individual at any age can be infected [6, 9, 32]. As such, there is a high likelihood of an individual having a NCD, HIV/AIDS, and comorbidity of NCD and HIV/AIDS at any age [3, 28, 33, 34]. Young adults are of particular interest because they are in the prime of their productivity, and usually the backbone of developing economies. If young adults are affected by NCD, and NCD‐HIV/AIDS comorbidity, productivity loss should be expected due to time off work [35].

NCDs and HIV/AIDS are both life‐long illnesses that are fatal. As such, the occurrence of a NCD and HIV/AIDS in an individual would prove to be more lethal [5, 36]. A critical point highlighted in studies that have discussed NCDs among people living with HIV is that due to antiretroviral treatment, people live longer and therefore are prone to NCDs [26, 37, 38]. Nevertheless, the NCD‐HIV/AIDS comorbidity is a cause for concern regardless of which illness, whether a NCD or HIV, afflicted an individual first [15, 33, 36, 39]. Hence, the need to study trends and forecasts, especially in SSA [2].

Malawi is one of the countries with growing NCD cases within a region with the most HIV/AIDS cases [2, 9, 11, 19, 20, 33, 40]. Therefore, Malawi has a high chance of having NCD‐HIV/AIDS comorbidity [17, 38, 41, 42]. However, there is limited data to assess the trends in NCD and NCD‐HIV/AIDS comorbidity [29, 37]. This study, therefore, provides insight into trends of NCDs and NCD‐HIV/AIDS comorbidity. It will also highlight the prominent NCD that requires more attention and resources. Thus, enabling progress monitoring and evaluation in achieving WHO and SDG targets in a data limited setting [2, 40, 43, 44].

The study will provide evidence to assist in developing policy, interventions, and responding to the evaluation of SDG3 targets. Furthermore, the study also contributes to the challenge of limited information on trends of NCDs and NCD‐HIV/AIDS comorbidity [37]. Therefore, this study aims to assess trends and provide forecasts in both NCD and NCD‐HIV/AIDS comorbidity to determine whether there is progress towards attaining SDG 3.3 and 3.4.

Despite the general trends and forecasts in NCDs and NCD‐HIV/AIDS comorbidity cases, this study's focus was on hypertension [45, 46, 47, 48]. The WHO's International Classification of Diseases (IDC), Centres for Disease Control and Prevention, and American Heart Association classification of hypertension as a NCD has been adopted in this study [45, 46, 47, 49, 50, 51]. Hypertension is a cause for concern due to the increase in the number of cases among other NCDs in the SSA [42, 48, 52]. Risk factors leading to hypertension still persist, especially in developing countries [53]. In addition, LMICs comprise two‐thirds of all hypertension cases, with the African region having a prevalence of 27% [47, 54]. As evidenced from the summary statistics, hypertension accounted for 67% of the NCD cases observed; hence, the study's focus on hypertension [55].

Determining NCD, NCD‐HIV/AIDS, hypertension, and hypertension‐HIV/AIDS case trends requires modelling methodologies that are able to predict and forecast. Machine learning (ML) models have been employed in NCD studies in predicting risk factors and proneness to a particular NCD [56, 57, 58, 59]. However, the ML models have not been employed in studying trends, leveraging their highly accurate predictive power compared to traditional models and conducting forecasts [60, 61, 62]. As such, this study employs ML models, specifically Extreme Gradient Boosting, in assessing the trends, making predictions, and forecasts for NCDs, NCD‐HIV/AIDS comorbidity, hypertension, and hypertension‐HIV/AIDS comorbidity cases.

## METHODS

### Study design

The study followed a retrospective study design where data was extracted from NCD patient Mastercards, which are paper‐based health cards used to record NCD patients' data and treatment history.

### Population and sample

The population comprised all NCD and NCD‐HIV/AIDS patients in the cities and districts in Malawi that had characteristics of the drivers of NCD risk factors. Such factors included urbanisation, development, and growing economic activity [3, 4, 63]. All the cities and districts with these characteristics were selected to be included in the study. Thus, four cities (Blantyre, Zomba, Lilongwe and Mzuzu) and seven districts (Mwanza, Neno, Mangochi, Salima, Mchinji, Nkhatabay and Karonga) were selected. From these cities and districts, only health facilities that were using patient Mastercards to record NCDs were included in this study. A total of 70 health facilities were included in the study, from which 30,686 patient records were extracted.

### Data

Patient level data ranging from July 2019 to December 2022 was extracted. The data consisted of: dates of diagnosis; NCD diagnosis; HIV/AIDS diagnosis; district; region; city/district locations; city/district designation; gender; and age. Data on 30,686 patients was extracted from the NCD Mastercards using a data extraction form that was designed and implemented in KoboToolBox. Android devices were used by trained fieldworkers who extracted the data for a 6‐month period from September 2022 to February 2023. The data was then downloaded from KoboToolBox in Excel format.

### Data management

Data management started with data preprocessing which involved formatting and cleaning the data by ensuring that the dates had the same format and variable values have uniform codes. Data preprocessing was done in a spreadsheet application, MacOS Numbers version 14.3.

Further preprocessing was done in R version 4.4.0 and RStudio 2024.04.2 + 764. The data also had some missing dates comprising 6.4% (*n* = 27,327) of the data. The data with missing dates was excluded from the analysis since the date was vital information for assessing trends. Data imputation was considered but resulted in the majority of dates being between July 2019 and December 2019. Pre‐July 2019 patient data was captured as new patients in July 2019, and most of the last half of 2019, which is also when new health facilities were being added to the mastercards programme. This inflated new patients' data, and therefore, in order to easily understand the trends of newly diagnosed cases, this data was trimmed from the analysis. Data transformation involved aggregating the data to weekly counts of NCD and NCD‐HIV/AIDS cases and scaling the data; further trimming off all the 2019 data also necessitated that the data was within an identical time frame of 52 weeks in a year and ranging from January 2020 to December 2022. A sample comprising 17,741 patient data was used for the analysis of the study after trimming the data.

### Data analysis

Analysis of data was conducted in R version 4.4.0 and RStudio 2024.04.2 + 764. Data analysis began with descriptive analysis using time series plots to summarise trends of all cases in general, by gender, age group, region, city/district location and city/district designation. Time series plots were also used for individual NCD cases combined with NCD–HIV/AIDS comorbidity cases to show their trend. Thereafter trends for the cases were segregated by specific NCD only cases and comorbidity cases which were then presented on the time series plot.

Data exploration was conducted by constructing a time series decomposition to aid in improved understanding of the behaviour of the time series and assessing the autocorrelation plots. This was done to determine seasonal lags, thereby aiding in defining features added to the model for prediction and forecasting [64] (Additional file 1).

Extreme gradient boosting (XGBoost) regression was used to train a weekly cases model to develop predictions and forecasts of hypertension and hypertension‐HIV/AIDS cases [65]. XGBoost modelling combines predictions from multiple decision trees to develop a strong predictive model [65, 66].

The objective function of XGBoost regression comprises the loss function and regularisation term given by

(1) $$L\left(\theta \right)=\sum_{i=1}^{n}L\left(y_{i},{\hat{y}}_{i}\right)+\sum_{k=1}^{K}\Omega \left(f_{k}\right)$$

where:

$L\left(y_{i},{\hat{y}}_{i}\right)$ is the loss function each number of weekly cases, i, which is the regression square error loss expressed as ${\left(y_{i}-{\hat{y}}_{i}\right)}^{2}$ and $\Omega \left(f_{k}\right)$ is the regularisation term for each individual tree, k, expressed as $\mathrm{\gamma T}+\frac{1}{2}\lambda \sum_{j=1}^{T}w_{j}^{2}$ defined as T is the number of leaves in tree k, $w_{j}$ is the weight of leaf j, γ penalises the number of leaves to control the complexity, λ controls weights through regularisation. Thus, the XGBoost regression is finally expressed as

(2) $$L\left(\theta \right)=\sum_{i=1}^{n}{\left(y_{i}-{\hat{y}}_{i}\right)}^{2}+\mathrm{\gamma T}+\frac{1}{2}\lambda \sum_{j=1}^{T}w_{j}^{2}$$

The gradient boosting framework aims at fitting a new weekly cases predictive model to the residuals of the previous weekly cases model in order to minimise the objective function using a gradient descent optimisation method [65]. XGBoost further optimises the gradient boosting technique by training the new weekly cases model through the second order approximation [65].

(3) $$L\left(\theta \right)=\sum_{i=1}^{n}\left[L\left(y_{i},{\hat{y}}_{i}\right)+g_{i}\left({\hat{y}}_{i}-{\hat{y}}_{i}^{\left(m\right)}\right)+\frac{1}{2}h_{i}{\left({\hat{y}}_{i}-{\hat{y}}_{i}^{\left(m\right)}\right)}^{2}\right]+\sum_{k=1}^{K}\Omega \left(f_{k}\right)$$

Upon convergence, a sum of all the predictions comprising all the trees is the model output that is used to make final predictions and forecasting for the next 8 years from 2023 to 2030 [65, 66]. The function for the model output and prediction is given by

(4) $$\hat{y}\left(x\right)=\sum_{m=1}^{M}\eta h_{m}\left(x\right)$$

where $h_{m}\left(x\right)$ is the *m*th tree prediction and M is the total number of trees.

The prediction and forecast trends were on: all cases (NCD and NCD‐HIV/AIDS cases); all hypertension cases (hypertension and hypertension‐HIV/AIDS cases); hypertension only cases; hypertension‐HIV/AIDS cases; all hypertension cases in young adults (hypertension and hypertension‐HIV/AIDS cases in young adults); hypertension only cases in young adults; and hypertension‐HIV/AIDS cases in young adults. These definitions apply to the data references in the manuscript while young adult refers to individuals aged 19–39 years old.

XGBoost regression using decision trees was employed to fit a model to the data and make predictions as well as forecasts using the xgboost library in R and RStudio [65, 67]. Features including day of the year, day, week, month, and year were engineered from the date object since ML models do not get an informative input from the date [68]. Additional features included trend since decomposing the data showed an upward trend with a slight curve. The slight curve prompted an additional feature of a quadratic trend, in addition to the log of the trend to account for the gradual growth in the number of cases. The autocorrelation plots showed seasonality in the last week of the year at lag52 which was included as a feature for the analysis. The tidyverse library was used to engineer the features required for the analysis.

The data used for the analysis was divided into training and testing sets with an 80/20 split to assess how well the model would perform on new data [69, 70]. The model performance on the test set can visually be seen from the figures and the MAPE evaluation metric [71, 72]. Furthermore, cross‐validation was employed to ensure generalisation of the model to an independent dataset [70, 73, 74, 75]. A five‐fold cross‐validation was used for the XGBoost regression model to prevent overfitting, selection bias, and enable generalisation to new data [73, 76, 77].

XGBoost requires a set of hyper‐parameters that are used to determine the best model to fit the data through five‐fold cross validation. Wider tuning ranges for all the hyper‐parameters were initially chosen through experimentation in a grid search method. For instance, ‘nrounds’ had a tuning range of 100–1000 with 100 increments. The tuning ranges were reduced as shown in Table 1, considering the hyper‐parameters selected by the all cases model. Table 1 shows hyper‐parameter tuning sets that were used.

**TABLE 1:** Hyper‐parameter tuning set values for the XGBoost models and their definition.

Hyper‐parameter	Tuning range used	Description
nrounds	100, 200, 300, 400, 500	Number of gradient‐boosted trees
max_depth	seq [10, 20]	Maximum depth of each tree
colsample_bytree	0.25, 0.5, 0.75	Subsample ratio of features used when constructing each tree
eta	0.025, 0.05, 0.75	Learning rate for each iteration while optimising the objective function
gamma	0.05, 0.5	Minimum loss reduction required for a new partition on a leaf node
min_child_weight	seq [1, 3]	Minimum sum of instance weight (hessian) needed in a child node
subsample	0.25, 0.5, 0.75	Ratio of the training data sampled for each tree

The specific hyper‐parameters values selected for each of the XGBoost models for the different subsets of data are shown in Table 2.

**TABLE 2:** Hyper‐parameter values selected for each of the XGBoost models for different subsets of data.

	Nrounds	max_depth	colsample_bytree	Eta	Gamma	min_child_weight	Sub‐sample
All cases	400	12	0.25	0.05	0.5	2	0.5
All hypertension and hypertension‐HIV/AIDS	100	13	0.25	0.05	0.05	2	0.5
Hypertension only cases	100	12	0.5	0.05	0.5	2	0.75
Hypertension‐HIV/AIDS comorbidity cases	300	11	0.5	0.75	0.05	2	0.75
All young adults hypertension and hypertension‐HIV/AIDS cases	100	20	0.5	0.05	0.05	1	0.25
Young adults hypertension only cases	200	11	0.25	0.25	0.05	1	0.25
Young adults hypertension‐HIV/AIDS cases	100	16	0.5	0.05	0.05	3	0.25

To determine the best model which fits the data, several metrics were used. The metrics used were coefficient of determination(*R* ^2^), root mean square error (RMSE), mean absolute percentage error (MAPE), and mean absolute error (MAE) [78].

*R* ^2^—measures the variation explained by the XGBoost regression model and a high value of *R* ^2^ implies a better model. Coefficient of Determination is given by the formula:

(5) $$R^{2}=1-\frac{\sum_{i=1}^{N}{\left(y_{i}-{\hat{y}}_{i}\right)}^{2}}{\sum_{i=1}^{N}{\left(y_{i}-\bar{y}\right)}^{2}}$$

Where $y_{i}$ is the actual observed value at time point $t.\hat{y}$ is the predicted value at time point t. $\bar{y}$ is the mean of y.

MAPE determines the accuracy of the model by calculating the sum of the average absolute value of percentage error between data values and forecasts for each time point. A small MAPE implies high accuracy. The formula for MAPE is as follows:

(6) $$\text{MAPE}=\frac{1}{n}\sum_{t=1}^{n}\left|\frac{A_{t}-F_{t}}{A_{t}}\right|$$

RMSE calculates the average squared errors between the data values and forecast values for each time point, and high accuracy of a model is determined by a lower RMSE value. The formula for this metric is:

(7) $$\text{RMSE}=\frac{1}{n}\sum_{t=1}^{n}{\left(A_{t}-F_{t}\right)}^{2}$$

MAE calculates the mean absolute difference between the actual data values and forecasts for each unit of time. This model evaluation metric also has to be lower for a model to be deemed accurate. The formula is:

(8) $$\mathrm{MAE}=\frac{1}{n}\sum_{t=1}^{n}\left|A_{t}-F_{t}\right|$$

where $A_{t}$ is the actual value at time‐point t. $F_{t}$ is the forecasted value at time‐point t. n is the number of data points in the time‐series.

It is worth noting that traditional statistical techniques under time series modelling (i.e., count time series, time series linear models, simple exponential model, ARMA and ARIMA) were used for trend analysis and forecasting; however, the white noise assumption, among others, was not satisfied. ML models have an upper hand in that they have the advantage of tuning hyperparameters, which enables obtaining the optimum model. This characteristic enables flexibility in ML models such as XGBoost [79].

## RESULTS

### Descriptive analysis

**FIGURE 1:**
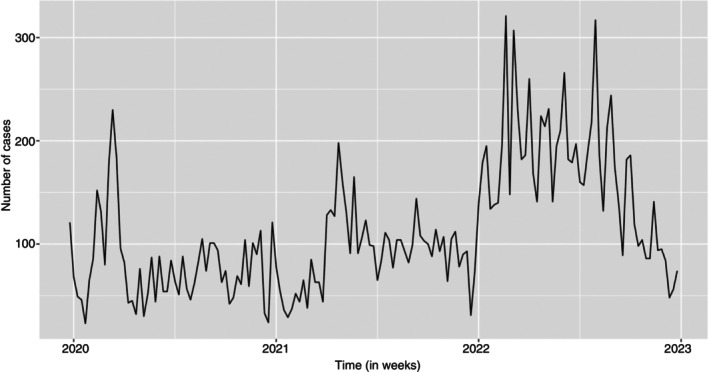
Time plot for all cases (NCD and NCD‐HIV/AIDS cases).

A summary of all cases was evaluated using a time series plot. Figure 1 shows that there were high counts of cases in the first quarter, which peaked at about 230 cases per week in March 2020. The count of cases fluctuated between 20 and 120 cases per week for the rest of 2020. The year 2021 follows a similar pattern, where the beginning of the year has high case counts and decreased towards the end of the year. The case counts went higher, peaking at about over 300 case counts per week around February, March, and then in July. Generally, there were higher case counts throughout the third year compared to the previous years, while the pattern still stayed the same.

The cases were summarised by socio‐demographic characteristics of the patients using time plots.

**FIGURE 2:**
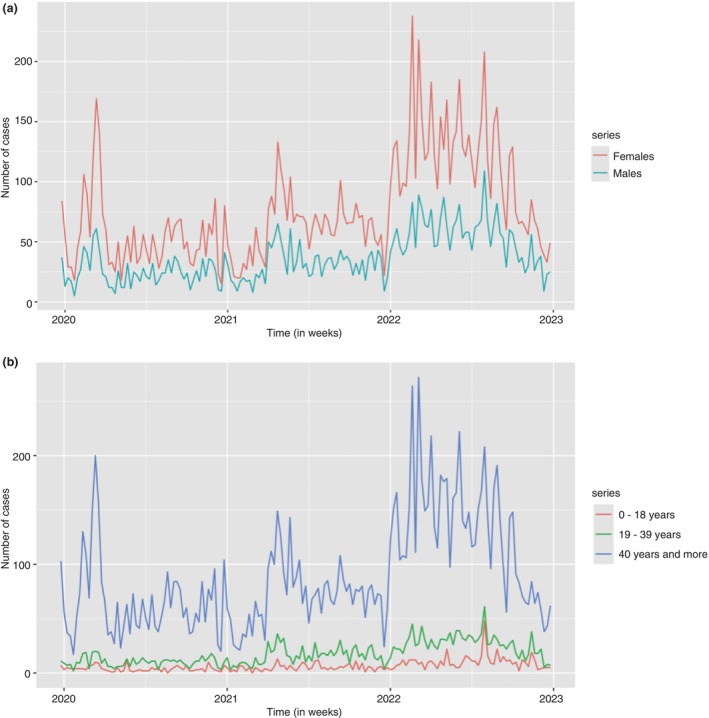
Time plots for cases socio—demographic characteristics.

Figure 2a is the gender time plot from which each gender basically follows the same pattern as the original data in Figure 1. The case counts for females are higher throughout the series, with high case counts in the first half of each year and the case counts lowering as the year progresses. The case counts for the males can be considered fairly consistent through the years, with a slight increase above 50 case counts per week in the year 2022.

The case counts were also summarised by the different age groups and Figure 2b shows the patterns of the cases for each age group. The 40‐year and above age group constitutes the greatest number of case counts. The number of cases ranging between 100 and 210 case counts per week was noticed in the early months of the first 2 years, which later escalated to more than 250 case counts in the final year. Thus, the number of case counts decreases as each year progresses, but the final year still has a high case count despite the decrease.

The age group 19–39 years has a lower number of case counts compared to the older age group, but follows a similar pattern in the flow of the case counts. The least case counts were observed for the 0‐ to 18‐year‐old age group, with some weeks having zero case counts.

**FIGURE 3:**
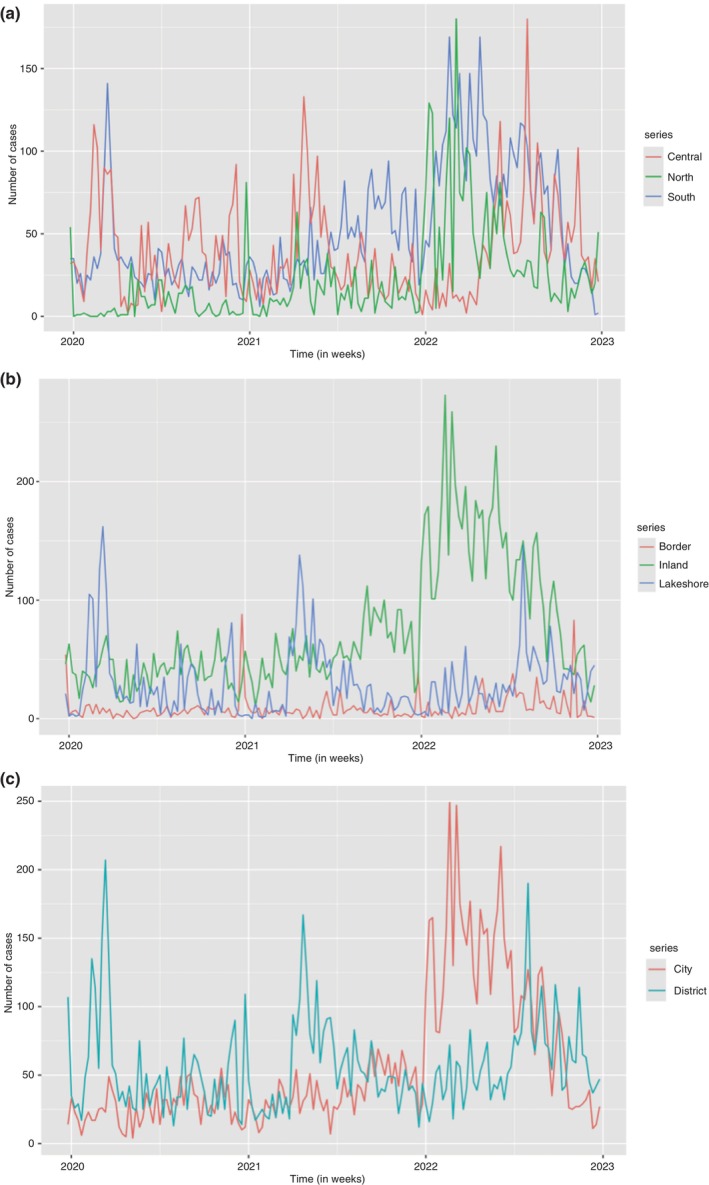
Time plots for different locations.

Three different types of locations were also used to summarise the NCD and NCD‐HIV/AIDS case counts. Data was summarised by region (North, Central, South), city/district designation (city, district), and city/district location (inland, border and lakeshore) as shown in Figure 3. There was not much distinction in the progression of case counts per week throughout the given time period for the Central and Southern Regions. The northern region has low case counts for years 2020 and 2021, but there is a rise in case counts in the year 2022, with the highest peak slightly above 175 case counts per week. In terms of city/district location, inland locations had a steady fluctuation of case counts for the first 2 years, with a sharp rise in case counts from the beginning of 2022 and a gradual decline as the year progressed. Lakeshore areas had some high numbers of case counts in the first halves of 2020 and 2021 within the months of March to May. Meanwhile in 2022 the peak in case counts was in the second half of the year, particularly in August; however, the beginning of the year had cases fluctuating below 50 cases per week. Border areas had the least number of cases, with some spikes in the number of cases for some weeks in December each year. Cities had case counts that were below 50 from 2020 to mid 2021 from where case counts began to rise and peaked at about 260 cases per week in March 2022. The rest of the year showed a gradual decline in the number of cases per week. In the first, second, and third quarters in 2020, 2021, and 2022 respectively, the districts had some weeks with case counts of 150 cases per week. Compared to the cities, the districts had slightly higher case counts per week in 2020 and 2021; however, cities led with about double the case counts in the first quarter of 2022.

Since most cases emanated from inland areas, which include all cities in Malawi, it can be concluded that cities contribute to the increasing trends of NCDs, HIV/AIDS, and NCD‐HIV/AIDS comorbidity. The districts account for most of the lake shore count cases and include one inland district, Neno.

**FIGURE 4:**
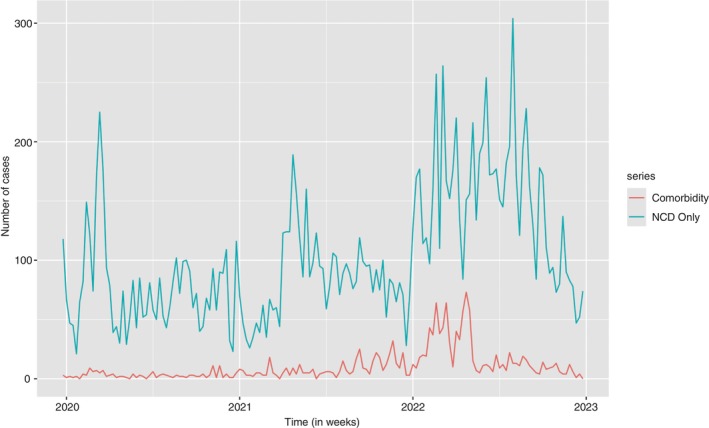
Time plot for NCD only cases and comorbidity cases.

The case counts were split into NCD only cases and NCD‐HIV/AIDS comorbidity cases, which were then displayed using a time plot (Figure 4). Most of the cases were NCDs, as seen by the high case counts for each week in the time plot. The first half of each year, 2020 and 2021, had some weeks with high case counts per week. The year 2022 had the case counts fluctuating higher from January to August, where the case count began to decline. There were some comorbidity cases which went to a maximum of about 20 cases per week from 2020 to mid 2021 where an increase in the number of case counts was noticed. A significant increase was also observed in the first half of the year 2022, then a drop in the number of case counts throughout the rest of the year.

**FIGURE 5:**
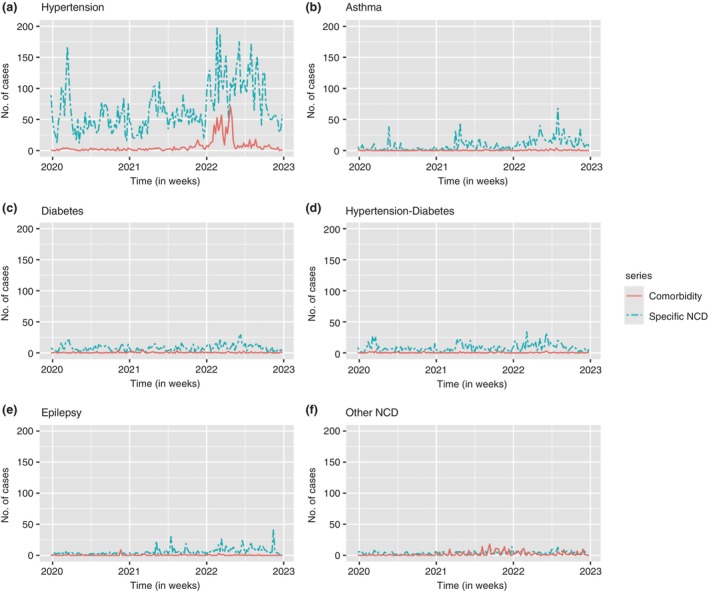
Aggregation of cases by specific NCD only and NCD‐HIV/AIDS comorbidity cases.

The cases were subset by the prominent NCDs observed in this study and summarised using time plots (Additional file 2). Figure 5a for hypertension shows cases progression for combined case counts for hypertension only and hypertension‐HIV/AIDS. The progression of hypertension case counts had most cases being recorded in the first half of each year and reducing to a constant fluctuation for the first 2 years between 20 and 100 case counts per week. The final year followed a gradual decline in the number of weekly case counts, with the number of cases averaging above 100 cases per week. A steep decline was seen in the last 3 months of the year 2022. The other NCD case counts showed low counts per week and were consistent such that the case counts averaged between zero and 30 cases per week, except for asthma in the third quarter of 2022.

The case counts shown in Figure 5 refer to the aggregation of cases by specific NCD only and NCD‐HIV/AIDS comorbidity cases. The cases were aggregated separately and plotted on the same time plot.

Specifically, hypertension only cases and hypertension–HIV/AIDS comorbidity cases were aggregated separately and shown in additional file 2, figure A. Hypertension only cases displayed similar fluctuations to combined cases in additional file 2, fig. A. High fluctuations in case counts were observed in the first half of each year, with a sharp increase in the last year. For the first 2 years, a consistent fluctuation in the case counts was observed, while the final year showed a gradual decline. The hypertension‐HIV/AIDS comorbidity cases were consistently averaging between zero and approximately 10 case counts per week, with a slight increase towards the end of year 2021. The most increase in comorbidity was noted in the first half of the year 2021.

The other specific NCD only case counts fluctuated consistently close to zero with an exception for case counts for asthma. Comorbidity case counts were mostly zero counts per week and began to increase towards the second half of 2022. A substantial increase in the case counts was observed in the first half of 2022 with higher fluctuations and a peak of about 75 case counts per week.

## MODELLING WEEKLY CASE COUNTS WITH XGBoost

Extreme gradient boosting model was used for predictions and forecasting. The model was able to make particularly good predictions on the training data and made fair predictions on the test data based on the MAPE metric. The MAPE metric and visual examination of the prediction data showed predictions were not significantly different from the actual data. The coefficient of determination was integral in understanding the amount of variation that was explained by the XGBoost regression model. The forecasts were reliable due to the good predictions made by the model. The forecasts were conducted from 2023 to 2030 to observe the progression of the cases and assess the progress towards achieving SDG3.

### General case counts per week

**FIGURE 6:**
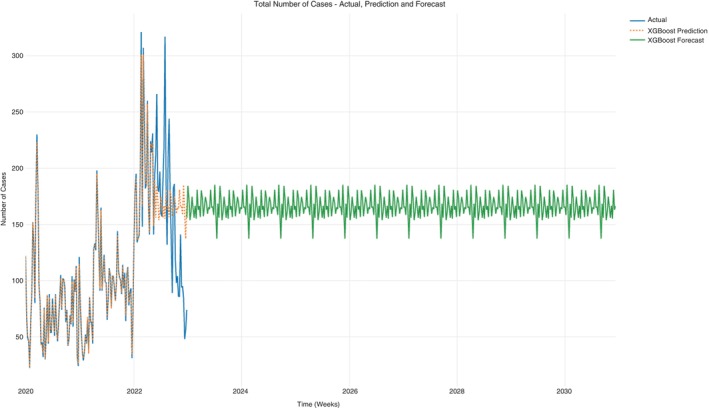
All cases time plot prediction and forecast.

The case counts per week for NCD and NCD‐HIV/AIDS comorbidity cases are shown in the time plot in Figure 6. The prediction shows a consistent progression in the case counts per week which on average had a minimum of 137 and a maximum of 185 case counts per week (Table 3).

**TABLE 3:** Average forecasts of the different weekly case counts.

	Average forecasted weekly case counts	
Model	Minimum	Maximum
All cases	137	185
Hypertension and hypertension‐HIV/AIDS cases	111	124
All hypertension only cases	92	115
Hypertension‐HIV/AIDS comorbidity cases	2	9
All young adults hypertension and hypertension‐HIV/AIDS cases	8	12
Young adults hypertension only cases	7	10
Young adults hypertension‐HIV/AIDS cases	2	3

### All hypertension cases

A subset on hypertension was taken from the dataset to evaluate the progression of the prominent NCD only.

**FIGURE 7:**
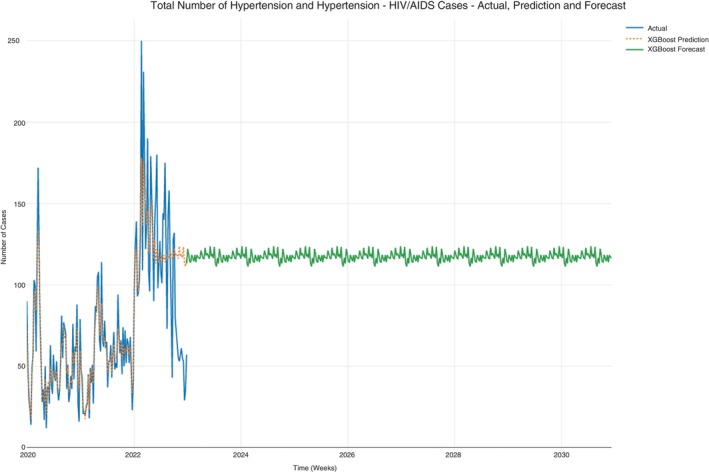
All hypertension cases (hypertension and hypertension‐HIV/AIDS cases).

**FIGURE 8:**
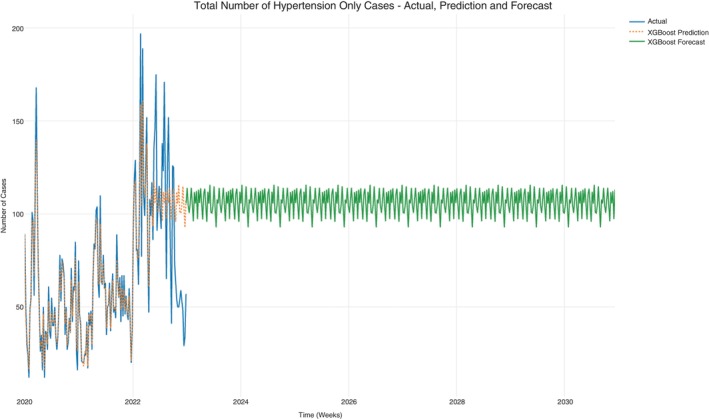
Hypertension only cases.

**FIGURE 9:**
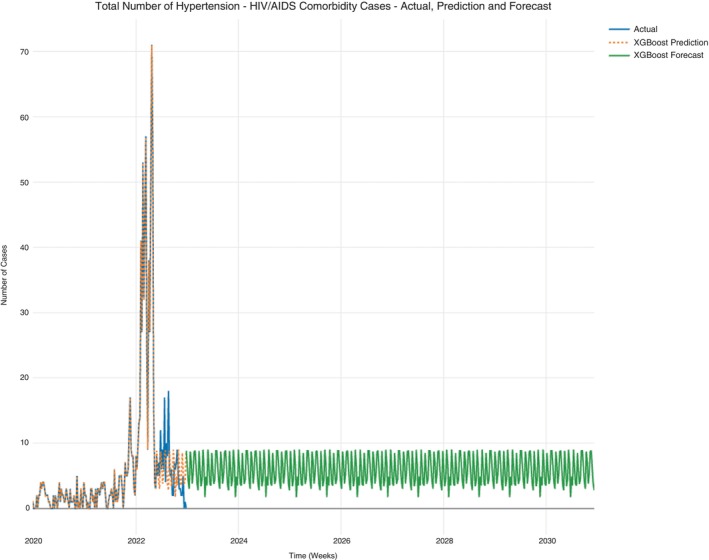
Hypertension‐HIV/AIDS comorbidity cases.

Case counts for hypertension and hypertension‐HIV/AIDS were subset from the data set since hypertension was the prominent NCD. The cases from this data set were further subset to hypertension‐only cases and hypertension‐HIV/AIDS cases. The prediction in Figure 7 also shows a consistent progression of all hypertension case counts per week that does not decline. This observation is similar for hypertension‐only case count projections (Figure 8) and the hypertension‐HIV/AIDS comorbidity case counts (Figure 9). The average projection ranges are shown in Table 3.

Further sub‐setting was conducted to extract hypertension case count for young adults (19–39 years) so that a model was developed to make predictions and forecasts specifically for this age group.

### All hypertension and hypertension‐HIV/AIDS in young adults

**FIGURE 10:**
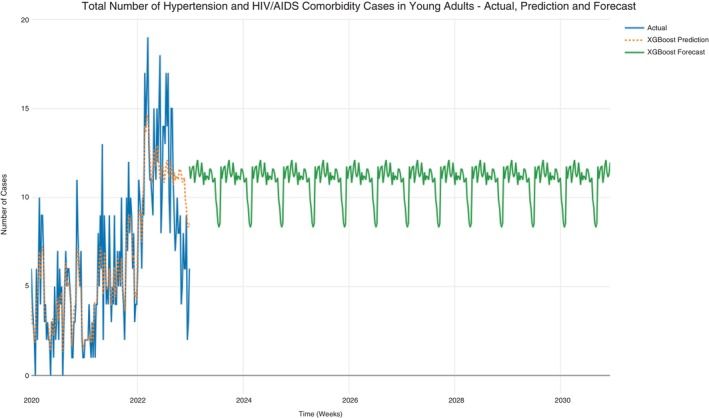
All hypertension and hypertension‐HIV/AIDS cases in young adults.

**FIGURE 11:**
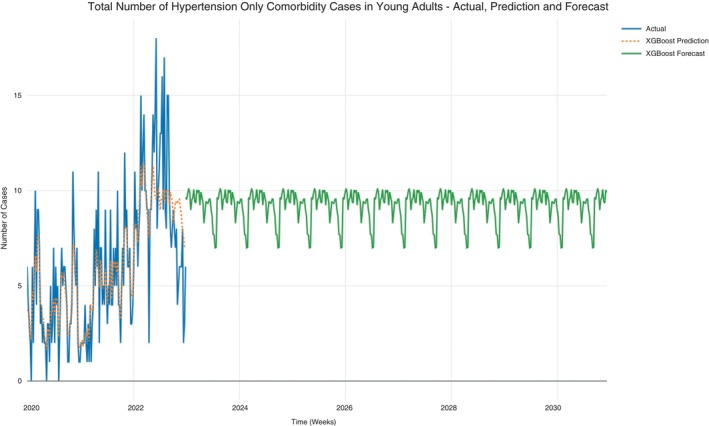
Hypertension only cases in young adults.

The hypertension data was further subset to constitute young adults only (19–39 years). Predictions and forecasts for case counts per week were conducted for hypertension and hypertension‐HIV/AIDS cases, hypertension only cases, and hypertension‐HIV/AIDS among young adults. Prediction for case counts was fair (MAPE = 0.528) and hence allowed forecasts to be conducted. The case counts in the original data ranged between 0 and 18 cases per week. In addition, the forecast progression had an average minimum of 8 and maximum of 12 case counts per week, with most cases subtending towards the maximum side (Figure 10). A steady, consistent, and uniform progression of the case counts was also observed from the forecast. Similar patterns were also observed for the hypertension only case counts for young adults (Figure 11).

**FIGURE 12:**
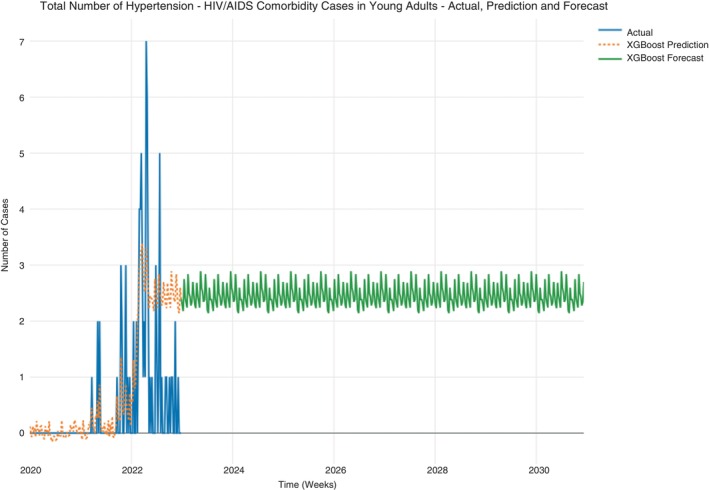
Hypertension‐HIV/AIDS cases in young adults.

The hypertension‐HIV/AIDS comorbidity case counts in young adults data ranged from 0 to 7 cases, and most of the cases were observed in 2021 and 2022, with the peak of the cases occurring in the first half of 2022. The predictions from the data were fairly good, as observed from the visual inspection and the other performance metrics, which meant the predictions were being derived from the data. The forecast for hypertension–HIV/AIDS comorbidity cases showed a consistent progression of case counts, which averaged a minimum of 2 cases and a maximum of 3 cases per week. The case counts did not show any decline in the cases up to 2030 (Figure 12).

The evaluation metrics obtained for the XGBoost models for each of the subsets of data are shown in Table 4. Since our data was split into training and test set, MAPE values have been presented for the test set and the full data set. The four evaluation metrics have been presented and the coefficient of determination was the primary metric of choice due to its interpretability, followed by the MAPE [75].

**TABLE 4:** Best model evaluation metrics.

	Evaluation metrics				
Model	*R* ^2^	MAPE (test)	MAPE (full)	RMSE	MAE
All cases	0.7181106	0.5034345	0.1202966	31.99255	23.29930
Hypertension and hypertension‐HIV/AIDS cases	0.7215202	0.6558063	0.2419238	26.02869	18.84745
All hypertension only cases	0.5356000	0.5897609	0.1787939	23.82909	18.30264
Hypertension‐HIV/AIDS comorbidity cases	0.8814355	Inf	Inf	5.192825	3.116439
All young adults hypertension and hypertension‐HIV/AIDS cases	0.5661601	0.5280879	Inf	2.544349	2.053412
Young adults hypertension only cases	0.4861663	0.4315534	Inf	2.394718	1.904670
Young adults hypertension‐HIV/AIDS cases	0.4065752	Inf	Inf	0.8825953	0.5050275

*Note*: When actual data has 0 values, the MAPE metric produces undefined values; hence the other metrics also presented. However, the model is not affected.

## DISCUSSION

The study aimed at examining current trends in NCDs and NCD‐HIV/AIDS comorbidity weekly case counts and conducting forecasts to assess the progress of Sustainable Development Goal 3.3 and 3.4. The study discusses trends in the socio‐demographic factors, NCDs only, and NCD‐HIV/AIDS comorbidity. Trends, predictions, and forecasts have also been discussed for the following: all cases; all hypertension cases; hypertension only cases; hypertension‐HIV/AIDS cases; all hypertension cases in young adults; hypertension only cases in young adults; and hypertension‐HIV/AIDS cases in young adults.

Females accounted for the high fluctuations in the NCDs and HIV/AIDS case counts as their time plot also resembles the all cases data time plot. Females were the most affected by the NCDs and NCD‐HIV/AIDS comorbidity, as evidenced in our previous study [55]. This is consistent with previous studies which reported within the region and globally that female adults were most at risk of NCD and NCD‐HIV/AIDS comorbidity as well as reported cases [8, 26, 37, 80].

NCDs have been known to mostly affect the older age group and this study shows similar findings where most cases were among the older age group [30, 33, 81]. On the other hand, HIV/AIDS does not discriminate on age; hence, any of the age groups could also be afflicted with HIV/AIDS [11, 32, 63]. Hence, there is a possibility of an individual having both a NCD and HIV/AIDS [36, 39, 82]. Particular attention must be given to the young adult age group (19–39 years) who were also affected by NCDs and HIV/AIDS [30]. This age group was of particular concern due to the cumulative weekly trends observed, which would affect their productivity and the economy [35]. The early age group, although with very low weekly cases, should not be neglected as NCDs and HIV/AIDS are both life‐long illnesses that could potentially affect them throughout their life [83].

There were no major distinctions in the progression of case counts among the different regions. Despite that, Inland and lakeshore locations had large weekly case counts of NCD and NCD‐HIV/AIDS cases. These locations are characterised by environmental drivers of increased NCD and HIV risk factors such as urbanisation, industrialisation, and high economic activities [4, 13]. This is alongside individual level factors like a sedentary lifestyle, diet changes, excessive use of tobacco and alcohol, urban poverty, promiscuous behaviour, and drug abuse [27, 63]. These modifiable risk factors are responsible for an increase in NCD and NCD‐HIV/AIDS comorbidity.

The southern region of Malawi has two cities, the commercial city of Blantyre and the former capital city, Zomba. Meanwhile, the central and northern regions have 1 city each, namely Lilongwe, the capital city, and Mzuzu respectively [84, 85]. In the south, Blantyre city had the majority of NCD and NCD‐HIV/AIDS cases compared to Zomba due to the large scale of urbanisation and industrialisation [85]. Furthermore, in comparing the southern region with the central and northern regions, there were higher trends from the southern region, which were partly attributed to the inclusion of Neno district cases. Although Neno is neither a border nor a lakeshore district, it was included in the study because it runs an integrated chronic care clinic, has a robust electronic data collection system, and all its health facilities are on the Mastercard programme. Hence, its high contribution to the large number of cases provides insight that cases from the other cities and districts were underreported [15, 20]. Nevertheless, the southern region still had higher weekly case counts, and these cases were underreported. This is considering the number of health facilities that were on the Mastercard programme and those that had not yet enrolled. The challenge of health facilities not enrolled in the Mastercard programme was also encountered in the central and northern regions. Hence, this implies NCD and NCD‐HIV/AIDS cases were underreported countrywide.

While weekly NCD and HIV/AIDS comorbidity case counts may not seem significantly high, they are still concerning since both non‐communicable diseases and HIV/AIDS are life‐long illnesses. This thereby implies an increasing trend in the cumulative case counts. Individually, NCDs and HIV/AIDS are deadly diseases which make having a comorbidity worse [86, 87]. The increase in comorbidity case counts is expected to continue rising as more people living with HIV are prone to develop a NCD as they age [3, 26, 39, 82, 88]. The risk of a double burden is not only to PLHIV, but an individual with an NCD is at risk of HIV/AIDS, especially in SSA where conditions for a comorbidity are present [3, 20, 38].

Hypertension is the most prominent NCD in Malawi and other countries, considering the number of studies that have been conducted on hypertension as well as hypertension comorbidity with other diseases [27, 30, 89, 90, 91]. Hypertension cases were the major contributor to the trends observed in the all cases data. As observed in the hypertension and hypertension‐HIV/AIDS comorbidity cases trends, there was a consistent fluctuation in the new weekly cases. The peaks observed may be attributed to new health facilities being added on the NCD Mastercards programme. The new weekly cases cumulatively imply an increasing trend as well as a forecast in the number of cases. Similar trends have been observed in countries within the region and Low‐ and Middle‐Income countries [2, 41]. This increasing trend in mortality was also observed in the Western Pacific region, with some imbalances across different countries [2, 49, 92]. Furthermore, South Africa, an upper‐middle‐income country, has had increasing trends in elevated blood pressure and blood sugar among people living with HIV, alluding to an increase in comorbidity [93, 94]. On the contrary, trends have decreased by 1.3% per year globally between 1990 and 2017. Specifically, high‐income countries have had a decrease in NCD premature mortality, accounting for a large proportion globally. HICs are on track to achieve SDG 3.4, even though the progress among the different countries is uneven [2, 92]. The main emphasis of this study is that there was an increasing trend in the cumulative new weekly cases and specifically hypertension and hypertension‐HIV/AIDS comorbidity cases. The increased number in hypertension cases and hypertension‐HIV/AIDS cases leads to less productive time [6]. The other NCDs are equally fatal and worthy of study, but this study focused on hypertension and hypertension‐HIV/AIDS comorbidity due to the large number of cases.

The forecasts for NCD only cases, NCD‐HIV/AIDS cases, hypertension only cases, and hypertension‐HIV/AIDS cases data showed a steady consistent progression of case counts which did not show any decline throughout the forecast period. Since NCDs are life‐long illnesses that cannot be directly passed from one individual to another, it is expected that the case counts should be consistent. The high fluctuations in the original data have been attributed to the recruitment of more health facilities to commence using NCD Mastercards. The projections for all the data and subsets (NCD only cases, NCD‐HIV/AIDS cases, hypertension only cases and hypertension‐HIV/AIDS cases) do not indicate any decline in the case counts in their forecast range. This implies that there will be no reduction in new cases if interventions are not implemented, hence no reduction of NCD fatalities by one‐third by 2030 [43]. This further indicates an increasing trend in the NCD cases as evidenced by the decomposition of the original data. Despite the notion that comorbidity is defined as having both NCD and HIV/AIDS regardless of which disease afflicted an individual first, PLHIV are most at risk [26, 37]. Hypertension was the most common NCD as well as its comorbidity with HIV/AIDS [81, 95, 96]. Other studies have also shown hypertension as a common NCD, hypertension comorbidity with other diseases, and hypertension among PLHIV [97, 98, 99].

There is a need for sensitisation on NCDs and HIV/AIDS risk factors in cities and districts with high and increasing cases so that the number of cases per week can be controlled and reduced. Furthermore, although the young adult age group did not seem to have many case counts of NCDs, NCD‐HIV/AIDS, hypertension and hypertension‐HIV/AIDS, the increasing trend was still a point of concern. This was because new cases were added every week and the number of cases continued to grow [21, 100]. There is therefore a need for targeted intervention for young adults which should include a strong sensitisation message that NCDs are no longer diseases for older people, alongside the danger of having both an NCD and HIV/AIDS.

## LIMITATIONS

A limitation of this study was that artificial intelligence methods such as computer vision and image recognition, for extracting data which may have been quicker and more robust, were not used. Nonetheless, to ensure data validity, the two trained fieldworkers extracted data from the Mastercards, where the first did the initial entry and the second validated the entered data. For further validation, a picture of the Mastercard, with the personal identification information masked, was captured into KoboToolBox. Another limitation was not using artificial intelligence techniques in determining the tuning of hyper‐parameters. This was mitigated by initially using wider ranges for the hyper‐parameters and diminishing the ranges upon identification of probable smaller ranges. A further limitation of the study was that we did not explore the impact of the COVID‐19 pandemic on the findings due to data availability constraints on NCDs and COVID‐19 interaction. The study was also not able to show trends in the new weekly cases considering interventions that were being carriedout. The intervention features led to some of the model assumptions not being significant when added to the model; hence, the exclusion of the intervention features in the final model.

The strength of the study is that it provides insight into trends and forecasts of NCD and NCD‐HIV/AIDS in an era where data on these diseases is limited. Furthermore, the study was conducted in 4 cities and 7 districts, which provides better insight to NCDs and NCD‐HIV/AIDS burden compared to studying one or two cities/districts.

## CONCLUSIONS

The original data on NCDs and HIV/AIDS showed some significant fluctuations in the case counts per week which have been attributed to the addition of health facilities to the NCD Mastercard programme. However, these did not have a significant effect on the XGBoost model forecasting. The forecasts depict a constant variation in the number of new weekly cases for both NCD and NCD‐HIV/AIDS comorbidity with consideration to the general cases, hypertension cases and hypertension‐HIV/AIDS cases in young adults' case trends. Although there was consistency in the variation of new weekly cases, the trends in the number of cases were on the increase as new weekly cases accumulated on the previous cases.

There is a need for intervention to control the number of new weekly case counts so that Malawi should achieve its health objectives in the Malawi Vision 2063 and subsequently make progress towards achieving SDG 3.3 and 3.4. Malawi is already behind in achieving NCD indicators, including data availability, which would aid in assessing progress. The NCD situation is probably worse than it is and highly likely underreported due to limited data. The rise in NCDs is occurring in a region where HIV/AIDS is a problem, hence a higher likelihood of comorbidity cases and underreporting of the current comorbidity cases.

At the current trajectory, Malawi may not achieve the objectives spelled out in its National Health Research Agenda, Malawi Vision 2063, and at a larger scale, Sustainable Development Goal number 3, if intervention measures are not promptly implemented.

## AUTHOR CONTRIBUTIONS

AK was responsible for data extraction; MK and AK conceived, designed, and planned the study; AK analysed and interpreted the data and wrote the first draft of the manuscript; SM and AM interpreted the data and wrote the final draft of the manuscript together with MK and AK. All authors have critically reviewed and approved the final version of the manuscript.

## FUNDING INFORMATION

The Malawi University of Business and Applied Sciences provided financial support for the study.

## CONFLICT OF INTEREST STATEMENT

There are no competing interests declared by the authors.

## Data Availability

Data from which aggregates for the analysis are confidential.
